# Effects of SARS CoV-2, COVID-19, and its vaccines on male sexual health and reproduction: where do we stand?

**DOI:** 10.1038/s41443-021-00483-y

**Published:** 2021-10-27

**Authors:** Sharon P. Lo, Tung-Chin Hsieh, Alexander W. Pastuszak, James M. Hotaling, Darshan P. Patel

**Affiliations:** 1 Division of Urology, Department of Surgery, University of Utah Health, Salt Lake City, UT USA; 2 Department of Urology, University of California San Diego, La Jolla, CA USA

**Keywords:** Diseases, Reproductive disorders

## Abstract

Since severe acute respiratory syndrome coronavirus 2 (SARS-CoV-2) was first discovered, there have been questions surrounding the effects of coronavirus disease 2019 (COVID-19), and more recently the COVID-19 vaccine, on men’s health and fertility. Significant research has been conducted to study viral tropism, potential causes for gender susceptibility, the impact of COVID-19 on male sexual function in the acute and recovery phases, and the effects of the virus on male reproductive organs and hormones. This review provides a recent assessment of the literature regarding the impact of COVID-19 and its vaccine on male sexual health and reproduction.

## Introduction

Since severe acute respiratory syndrome coronavirus 2 (SARS-CoV-2) was first detected in December 2019 in Wuhan, China, there have been over 186,000,000 confirmed cases and over 4,000,000 deaths worldwide attributed to coronavirus disease 2019 (COVID-19) (https://coronavirus.jhu.edu/). Initially, very little was known about SARS-CoV-2 and COVID-19, but the medical and research community responded accordingly with a tremendous volume of research within a short period of time to better understand the virus and disease to offer better strategies for diagnosis, treatment, and prevention. This ultimately led to the development of multiple effective vaccines in under a year, an impressive and unprecedented accomplishment. Since the first vaccine was authorized by the FDA for emergency use in the United States on December 11, 2020; about half of the US population have been fully vaccinated (https://coronavirus.jhu.edu/).

As the pandemic continues to evolve with emerging variants and a growing volume of scientific literature, it is important for healthcare providers, policymakers, the media, and the general public alike to stay up to date on the latest literature. In the realm of men’s health, persistent questions remain regarding viral tropism, gender susceptibility, impact on semen parameters, hormones, sexual health, and the impact of vaccine on fertility. Our goal is to provide an update on the most recent literature.

### SARS-CoV-2 and viral tropism

SARS-CoV-2 is a single-stranded RNA virus of the coronavirus family. It is comprised of a nucleoprotein around an RNA genome, a viral envelope, and a spike protein. The spike proteins play a crucial role in receptor recognition, cell attachment, and fusion during viral infection [1]. Entry into host cells is facilitated by the viral spike protein and host cell receptor angiotensin-converting enzyme 2 (*ACE2*) [1–4]. Once the spike protein binds to *ACE2*, transmembrane protease serine 2 (*TMPRSS2*) on the host cell surface primes the spike protein and other cellular proteases to cleave the spike protein into two subunits. This then allows for viral entry and release of viral RNA so that viral genome replication and transcription can begin [2]. Both *ACE2* and *TMPRSS2* are required for viral entry into host cells [2].

SARS-CoV-2 is known to affect certain cell types based on their expression of *ACE2* and *TMPRSS2*. Once transmitted to an individual, replication of SARS-CoV-2 begins within the type II pneumocytes of the airway epithelial cells [5, 6]. Nasal secretory cells, absorptive enterocytes, and vascular endothelial cells are also affected [5–7]. *ACE2* is highly expressed in the testis, in both Sertoli and Leydig cells, as well as spermatogonia [8–12]; however, they are not known to express *TMPRSS2*. The body’s immune response to SARS-CoV-2 may also affect male reproduction in ways similar to those seen in other viral illnesses (i.e., mumps) [13].

In a study by Achua *et al*, testis tissue was collected from autopsies of COVID-19 positive (*n* = 6) and negative men (*n* = 3) [14]. The samples were stained with hematoxylin and eosin (H&E) and subjected to immunofluorescence for *ACE2* expression. Three of the COVID-19 positive biopsies had normal spermatogenesis while the other three had impaired spermatogenesis. The immunofluorescent stain slides from the six COVID-19 positive men demonstrated a direct association between increased quantitative *ACE2* levels and impairment of spermatogenesis. Tissues from four COVID-19 positive autopsy cases were also imaged with transmission electron microscopy (TEM). TEM showed the COVID-19 virus in the testis tissue of one of the cases. This study suggested that the testes may be a target of COVID-19 infection due to its high expression of *ACE2*; however, these findings are limited as they are based on a very small sample size with high risk of bias given the non-blinded nature of the study. Also, all three of their controls showed impaired spermatogenesis.

### Sex susceptibility to COVID-19

Although there are no good data examining the difference in incidence of COVID-19 between men and women, more men than women have died of COVID-19 in 41 of 47 countries and the overall case-fatality ratio is ~2.4 times higher among men than women [15–18]. Epidemiologic studies suggest a significant male sex susceptibility for more severe COVID-19 symptoms [18, 19]. There are a couple proposed theories as to why there are sex differences in COVID-19 outcomes. First, the gene for *ACE2* is located on short arm of the X chromosome. In females, one of the two X chromosomes is silenced causing condensation of the X chromosome into a Barr body; but some genes, particularly those on the short arm, escape this inactivation [20, 21]. This increased expression of *ACE2* in females may be protective against more severe COVID-19 symptoms as viral saturation is less likely to occur and *ACE2* regulates the renin-angiotensin system, which protects against vascular compromise and severe organ damage [21]. Next, lower circulating levels of androgens in females are thought to contribute to lower expression of *TMPRSS2* on host cell membranes and downregulation of this receptor [22]. The transcriptional promoter for *TMPRSS2* is androgen-responsive and was initially described in the context of *TMPRSS2-EGR* fusion gene and prostate cancer [22].

### Impact of SARS-CoV2 on reproduction

A number of studies have examined the presence of SARS-CoV2 virus in semen (Table 1) [23–26]. In almost all studies, no viral particles were discovered in the semen of men who were acutely infected or those who were recovering/recovered [23–34]. In only one study, the virus was detected in 6 out of 38 actively infected patients [35] while in another there were viral particles discovered in one man’s semen sample [36]. Overall, based on the current literature, it is unlikely that SARS-CoV-2 can be transmitted through the semen. This is not as important during acute infection since a person’s respiratory droplets would be easily transmissible, but it is important with regards to sperm cryopreservation or for intrauterine insemination or in vitro fertilization using ejaculated sperm in recovered men.

**Table 1:** Presence of Virus in Semen of Men Acutely Infected with COVID-19 and Recovered from COVID-19.

	Kayaaslan [23] *n* = 16	Best [24] *n* = 30	Ruan [25] *n* = 74	Ma [26] *n* = 12	Pan [27] *n* = 34	Holtmann [28] *n* = 34	Song [29] *n* = 13	Paoli [30] *n* = 1	Guo L [31] *n* = 23	Pavone [32] *n* = 9	Li D [35] *n* = 38	Gacci [36] *n* = 43	Temiz [33] *n* = 20	Li H [34] *n* = 23
Acutely infected	ND	ND				ND	ND		ND		Detected in 4 of 15 patients		ND	ND
Recovered		ND	ND	ND	ND	ND	ND	ND	ND	ND	Detected in 2 of 23 patients	Detected in one patient’s semen sample, rest ND	ND	

*ND* not detected.

With regards to sex hormones, there have been mixed findings (Table 2) [25, 26, 33, 37–40]. Two studies found that there were no statistically significant differences in LH levels between patients who had had COVID-19 and controls [31, 37], three studies found that LH was significantly increased in patients who had had COVID-19 compared to controls [26, 33, 38], one study found that LH was significantly decreased in patients who had had COVID-19 versus controls [39]. One study did not compare patients who had recovered from COVID-19 to controls but found that the mean LH level was 3.95 mIU/mL, which is within the normal range [25]. With regards to testosterone, four studies found no statistically significant difference between patients who had COVID-19 and healthy controls, although median time from confirmed infection was inconsistently reported [26, 31, 37, 39]. Two studies found that men with COVID-19 had significantly lower testosterone levels when compared to controls [33, 38]. And again, one study did not compare patients who had recovered from COVID-19 to controls but found that the mean testosterone level was 3.65 ng/ml, which is within the normal range [25]. It is not clear if the increases in LH and decrease in testosterone are related to febrile illness or from the direct effects of SARS-CoV2 on testicular cells, but Temiz did show that the decrease in testosterone during active infection seems to be temporary and there is at least partial recovery after treatment and Kadihasanoglu found that there was a difference in LH and testosterone level in those with COVID-19 versus non-COVID-19 upper respiratory tract infections [33, 38].

**Table 2:** Sex hormone levels of men with COVID-19.

	Ruan [25] *n* = 66	Ma [26] *n* = 12	Temiz [33] *n* = 20	Okcelik [37] *n* = 24	Kadihasanoglu [38] *n* = 89	Xu [39] *n* = 39	Guo TH [40] *n* = 41
LH	Not compared to controls. Mean of patients recovered from COVID-19 was 3.95 mIU/mL, SD 1.63	Significant increased (6.36 mIU/mL vs 3.38 mIU/mL, *p* < 0.0001)	Statistically significant difference between controls and COVID-19 patients before treatment and COVID-19 patients after treatment using Kruskal-Wallis test (2.98 IU/L vs 3.22 IU/L vs 4.46 IU/L, *p* = 0.04)	No statistically significant difference between those who tested negative for COVID-19 and positive for COVID-19 (5 mIU/ml vs 5.5 mIU/ml, *p* = 0.62)	Statistically significant difference between patients with COVID-19, patients with non-COVID-19 respiratory tract infection and age-matched controls (5.67 U/L vs 5.39 U/L vs 4.1 U/L, *p* = 0.0002)	Statistically significant decrease between patients with COVID-19 and controls (5.519 mIU/ml vs 8.051 mIU/ml, *p* < 0.05)	No statistically significant difference (3.6 U/L vs 3.6 U/L, *p* = 0.1903)
Testosterone	Not compared to controls. Mean of patients recovered from COVID-19 was 3.65 ng/ml, SD 1.19	No statistically significant difference (3.97 ng/ml vs 4.43 ng/ml, *p* = 0.1886)	Statistically significant difference between controls and COVID-19 patients before treatment and COVID-19 patients after treatment using Kruskal-Wallis test (2.90 ng/ml vs 1.13 ng/ml vs 2.26 ng/ml, *p* = 0.02)	No statistically significant difference between those who tested negative for COVID-19 and positive for COVID-19 (11.52 mmol/L vs 12.29 mmol/L, *p* = 0.56)	Statistically significant difference between patients with COVID-19, patients with non-COVID-19 respiratory tract infection and age-matched controls (185.52 ng/dL vs 288.67 ng/dL vs 332 ng/dL, *p* < 0.0001)	No statistically significant difference (3.932 ng/ml vs 3.838 ng/ml)	No statistically significant difference (3.6 ng/ml vs 3.5 ng/ml, *p* = 0.93336)

The studies examining the effect of COVID-19 on semen parameters are summarized in Table 3 [24–26, 28, 31, 33, 34, 36, 40]. Most studies showed a statistically significant decrease in semen concentration, total sperm count, and total motile count [24–26, 28, 35, 40]. One study found that semen parameters returned to the normal range after an average of 32 days after COVID-19 diagnosis [31] and one found that there was no statistically significant difference in semen parameters between controls, those who were actively infected, and those who had been treated for COVID-19 [33]. One study showed that the severity of the infection- whether the patient required no hospitalization, hospitalization, or ICU admission- was inversely correlated to semen parameters [36]. Again, unclear if differences are related to febrile illness or from the direct effects of SARS-CoV2 on the cells involved in gametogenesis as it is has been shown that viruses that cause febrile illness; such as chickenpox, influenza, and SARS; are associated with decreased sperm count and motility and abnormal morphology [40–42].

**Table 3:** Semen Parameters of Men with COVID-19.

	Best [24] *n* = 30	Ruan [25] *n* = 55	Ma [26] *n* = 12	Holtmann [28] *n* = 34	Guo L [31] *n* = 23	Gacci [36] *n* = 43	Temiz [33] *n* = 20	Li H [34] *n* = 23	Guo TH [40] *n* = 41
Semen concentration	Statistically significant decrease amongst actively infected men vs COVID-19 negative controls (11.5 × 10^6/ml vs 21.5 × 10^6/ml, *p* = 0.0048)	Decreased amongst men who had recovered from COVID-19 vs age-matched healthy controls (66.41 × 10^6/ml vs 81.34 × 10^6/ml, *p* = 0.0428)	66.7% had normal sperm parameters and low DFI. 33.3% had low sperm motility with higher sperm DFI.	Statistically significant decrease between mild COVID-19 and moderate COVID-19, and between control and moderate COVID-19	Within normal range after an average of 32 days after diagnosis	No statistically significant difference between pts who were managed with no hospitalization, hospitalization, and ICU admission (65.8 × 10^6/ml vs 17.8 × 10^6/ml vs 0.0 × 10^6/ml, *p* = 0.215)		39.1% showed sperm concentration <15 × 10^6/ml, significantly decreased compared to age-matched controls, mean ratio of 0.29 (*p* = 0.008)	Decreased amongst men who had recovered from COVID-19 vs control (49.6 × 10^6/ml vs 86.8 × 10^6/ml, *p* = 0.0115)
Total sperm count	Statistically significant decrease amongst actively infected men vs COVID-19 negative controls (12.5 × 10^6 vs 59.2 × 10^6, *p* = 0.0024)	Decreased amongst men who had recovered from COVID-19 vs age-matched healthy controls (197.40 ×10^6 vs 261.40 ×10^6/ml, *p* = 0.0211)				Statistically significant difference between pts who were managed with no hospitalization, hospitalization, and ICU admission (133.25 × 10^6 vs 38.1 × 10^6 vs 0.0 × 10^6, *p* = 0.021)	No statistically significant difference between controls, COVID-19 patients before treatment, and COVID-19 patients after treatment (48 × 10^6 vs 67.4 × 10^6 vs 69.9 × 10^6, *p* = 0.93)		Decreased amongst men who had recovered from COVID-19 vs control (148.9 × 10^6 vs 226.8 × 10^6, *p* = 0.0322)
Total motile count		Decreased amongst men who had recovered from COVID-19 vs age-matched healthy controls (48.89% vs 56.38%, *p* < 0.0001)					No statistically significant difference between controls, COVID-19 patients before treatment, and COVID-19 patients after treatment (18.68 × 10^6 vs 27.97 × 10^6 vs 23.54 × 10^6, *p* = 0.69)		Decreased amongst men who had recovered from COVID-19 vs control (66.1 × 10^6 vs 122.0 × 10^6, *p* = 0.0280)

*DFI* DNA fragmentation index.

### Impact of SARS-CoV2 on sexual function

There have been several studies investigating the relationship between COVID-19 and erectile dysfunction (ED). It has been proposed that the effects of COVID-19 on the cardiovascular system (i.e., acute cardiac injury, myocarditis) and central nervous system lead to decreased blood supply to the genitalia which can lead to ED [43–45]. Vascular integrity is also necessary for erectile function and endothelial dysfunction associated with COVID-19 is likely to affect the fragile vascular bed of the penis, resulting in impaired erectile function [45]. Pulmonary fibrosis associated with ARDS impairs physiologic lung mechanisms, reducing the pulmonary gas exchange and therefore impairing oxygen saturation. Hypoxia impairs erectile function as oxygen is one of the substrates required for synthesis of nitric oxide (NO) by NO synthase and NO activates guanylate cyclase in endothelial cells which results in increased concentrations of cyclic guanosine monophosphate, which in turn induces relaxation of vascular smooth muscle cells of the penile corpora cavernosa [45]. One study looked at the histopathological features of penile tissue of patients who had recovered from symptomatic COVID-19 infection and subsequently developed severe ED and underwent surgery for penile prosthesis. They found that extracellular SARS-CoV2 viral particles were found near the penile vascular endothelial cells and there was decreased endothelial NO synthase expression in the corpus cavernosum [46]. This study is limited by the fact that the sample size was very small (two patients) and there was no objective quantification of ED before and after SARS-CoV-2 infection.

Libido can be affected by biochemical, anatomical, and psychosocial factors. With COVID-19 causing one of the world’s largest economic crises and its significant effect on the well-being of individuals; some stress factors, such as domestic isolation, lack of movement and social contact, loss of jobs and economic problems, supply bottlenecks, limited health, and psychosocial care, and fear of and confrontation with infection and death, characterize life worldwide during the pandemic and all these factors have an effect on libido and sexuality [47]. Sexual activity is closely associated with mental and psychological health, so it is unsurprising that sexual desire and frequency have declined in both genders [45, 48]. Early in the pandemic, it was reported in a study from China that sexual frequency had decreased in 37% of those surveyed, and 44% reported a decrease in the number of sexual partners [47]. Interestingly a study from Bangladesh, India, and Nepal suggested minimal change in sexual activity and perhaps even an increase in frequency in a small subset [49].

Some have proposed that with lockdown restrictions and social isolation, there have been fewer opportunities for casual sexual meetings but increased frequency of sexual intercourse among couples in stable sexual relationships [50]. In a study of 967 young Chinese individuals, due to the COVID-19 pandemic and related containment measures, 22% of participants reported a decrease in sexual desire; 41% experienced a decrease in the sexual intercourse frequency; 30% reported an increase in the frequency of masturbation; 20% reported a decrease in alcohol consumption before or during sexual activities, and 31% reported a deterioration in partner relationships during the pandemic [51]. A study conducted in Italy involving 1576 participants showed that there was a significant decline in mean well-being scores during the quarantine compared to before, and there was a positive correlation between well-being scores and the number of sexual intercourse [52]. The mean number of sexual intercourse decreased significantly during quarantine with the main reasons reported as poor privacy and lack psychological stimuli [52]. Almost three-quarters of participants did not report sexual desire reduction and a positive association between sexual desire and sexual intercourse during the quarantine was found, but they also found that men presented lower sexual desire during the quarantine than women [52]. The fear of COVID-19 transmission has also had an effect on the sexual behavior of men, as seen in a study of 536 men in Turkey, in which 23.9% respondents stated that they had a fear of transmitting COVID-19 to their partner during sexual intercourse. While the number of weekly sexual intercourse of the participants before the pandemic was 2.34 ± 1.35, this decreased to 1.54 ± 1.45 during the pandemic period (*P* = 0.001) [53]. Finally, confinement as a result of lockdowns or illness itself can both be sources of stress, and this psychological suffering may exacerbate preexisting subclinical erectile dysfunction [45, 54].

### Impact of vaccines on men’s health and reproduction

There are currently three COVID-19 vaccines that have received emergency use authorization in the U.S.: Pfizer BioNTech, Moderna, and Janssen/Johnson & Johnson. The Oxford-AstraZeneca vaccine has been approved in the UK, but not the U.S. The Pfizer BioNTech and Moderna vaccines are mRNA vaccines, which are composed of fragments of mRNA that are taken up by immune cells, which transcribe the mRNA into spike proteins, the same ones that are found on the surface of SARS-CoV-2, which leads to immune recognition of these proteins and creation of antibodies against these proteins. These mRNA vaccines do not contain live viruses or use a viral vector. The Janssen/Johnson & Johnson and Oxford-AstraZeneca vaccines use more traditional viral vector-based technology, combining the piece of SARS-CoV-2 DNA that codes for the spike protein and combines it with disabled adenovirus to deliver it to immune cells. To date, there have not been any studies that compare the two vaccine technologies in terms of their impact on fertility, semen parameters, or sex hormones.

There have been concerns among the public about the impact of vaccines on sperm and infertility, mostly driven by vocal conspiracy theorists. Some of this fear stems from lack of understanding about newly developed mRNA-based vaccines and the misconception that spike proteins can allow the virus to enter gametes (and other adult cells) and alter the DNA. There has also been a focus on the fact that pregnant women were excluded from both studies and men and women of reproductive potential in the studies were required to utilize a highly effective method of contraception or remain abstinent [55, 56]. This was related to the strict protocols required for clinical trials, not due to concerns that the vaccines would be unsafe in pregnancy or affect fertility/offspring.

The most common side effects reported in both studies were injection side pain/redness/swelling, fatigue, headache, fevers, and chills. Serious side effects were rare but included shoulder injury related to vaccine administration, right axillary lymphadenopathy, paroxysmal ventricular arrhythmia, right leg paresthesia, and Bell’s palsy [55, 56]. There were no reported sexual and urologic side effects. Another study found that out of 15,785 adverse events reported to the FDA Vaccine Adverse Event Reporting System related to the Pfizer-BioNTech and Moderna vaccine, only 0.7% (*n* = 113) described urologic symptoms [57]. They included lower urinary tract symptoms (*n* = 34, 22%), hematuria (*n* = 22, 14%), urinary infection (*n* = 41, 26%), skin and/or soft tissue (*n* = 16, 10%), and other (*n* = 43, 28%). Only 18% (*n* = 20) of the adverse events reports described isolated urologic symptoms. None reported symptoms related to erectile function, ejaculatory function, or sexual function.

There is currently no evidence that the vaccine can cause infertility in men or women, damage to the placenta, or lead to miscarriages [58–63]. The ASRM states in their COVID-19 Task Force guidance document that because the COVID-19 mRNA vaccines are not composed of live virus, they are not thought to cause an increased risk of infertility, first or second-trimester loss, stillbirth, or congenital anomalies [64]. A small prospective study of 45 men showed that there were no significant decreases in any sperm parameters amongst this cohort of healthy men before and after 2 doses of a COVID-19 mRNA vaccine and that of the 8 men in the study who were oligospermic before the vaccine, none had further decline in sperm concentration [65]. They concluded that because the vaccines contain mRNA and not the live virus, it is unlikely that the vaccine would affect s parameters. Another study compared sperm parameters in 43 male patients undergoing IVF before and after receiving the Pfizer BioNTech mRNA vaccine [66]. They found that there were no differences in parameters; including volume, concentration, and total motile count; after vaccination. Of note, this study has not yet been peer-reviewed or published. Two other studies also found that COVID-19 vaccination does not impact male or female fertility or fertility treatment outcomes [67, 68].

Based on all currently available data, the vaccines are safe and there is no evidence to suggest any negative impact on fertility or sexual health. It is more likely that COVID-19 itself would have more long-term implications compared to the vaccine.

## Conclusions

While much has been published regarding SARS-CoV-2 and COVID-19 in the relatively short period of time since the disease was first detected and the cause identified, most studies are limited by their short duration and small sample sizes. Furthermore, there are significant conflicting data, likely related to the small sample sizes and limited diversity of the samples from which they were collected. With a few exceptions, most studies are based on retrospective data. Future studies should further examine the long-term effects of COVID-19 and its vaccines on men’s health and sexual function as well as the impacts it may have on their offspring. Initially, very small cohort studies helped characterize unique patient populations affected by COVID-19. These research efforts were essential in providing important data where no data previously existed. But caution should be exercised when it comes to sensationalized media coverage of COVID-19 research without critical assessment of scientific quality, and journals that are quick to publish low-quality research. As the pandemic evolves, we urge researchers to prioritize the conduct of scholarly investigation that has high scientific value to guide public health efforts.
